# Proposal of the optimal numbers of examined and positive lymph nodes to the 8th edition of American Joint Committee on Cancer (AJCC) staging for 758 patients with distal cholangiocarcinoma

**DOI:** 10.1371/journal.pone.0234464

**Published:** 2020-06-16

**Authors:** RuiYang Wu, Gang Zhang, Jiao Feng, Liang Zhang, ZhiMing Yang

**Affiliations:** 1 Department of Vascular Surgery, The First Affiliated Hospital of Chengdu Medical College, Chengdu, China; 2 Department of General Surgery, Sichuan Provincial Hospital for Women and Children, Chengdu, China; Mayo Clinic in Arizona, UNITED STATES

## Abstract

**Introduction:**

The American Joint Committee on Cancer (AJCC) recommended retrieval of at least 12 lymph nodes and firstly classified N category by the number of positive lymph nodes (PLNs) for Distal Cholangiocarcinoma (DCC).

**Objective:**

The end of this cohort study was to explore the optimal cut-off values of the number of examined lymph nodes (ELNs) and PLNs to better stratify patients by utilizing a population-based database.

**Methods:**

A number of 758 patients with DCC from the Surveillance, Epidemiology, and End Results (SEER) database were enrolled in the study and comparing by the survival analysis.

**Results:**

Survival analysis found that patients with ELNs < 5 had a lower 3-year disease-specific survival rate than ELNs ≥ 5 in N0M0 cohort (35.3% vs. 53.0%, P = 0.001) and in M0 cohort (42.7% vs. 32.8%, P = 0.006); survival curves between patients with ELNs < 12 and ELNs ≥ 12 were overlapped in N0M0 cohort (P = 0.256) and in M0 cohort (P = 0.233). Among patients with ELNs ≥ 5, using the optimal cut-off value of the number of PLNs (0, 2) could accurately stratify patients, but the recommendation of the number of PLNs (0, 3) by the AJCC could not.

**Conclusions:**

This study recommended examining at least 5 lymph nodes and defining PLNs = 1–2 as the N1 category and PLNs ≥ 3 as the N2 category, which may better stratify distal cholangiocarcinoma patients and improve the accuracy of the eighth edition AJCC staging.

## Introduction

Distal Cholangiocarcinoma (DCC) was an epithelial cell malignancy arising from the origin of the cystic duct to ampulla of vater [1], accounting for approximately 41.0–44.1% of cholangiocarcinoma [2–4]. And its incidence has gradually increased in recent year [5]. Although pancreaticoduodenectomy was the only potential treatment that can cure DCC patients with the resectable disease [6], the prognosis of patients with DCC after surgical resection was still poor, and the 5-year overall survival rates varied from 16–39.5% [7–10].

Adequate lymph node dissection could not only reduce the risk of tumor recurrence, but also improve the accuracy of staging. The number of lymph nodes retrieved could provide additional prognostic value [11]. Several articles reported lymph node status was one of the most important independent predictors for DCC patients with the resectable disease [2, 12]. Thus, American Joint Committee on Cancer (AJCC) recommended that at least 12 lymph nodes be retrieved, whereas the minimum number of examined lymph nodes examined (ELNs) has not yet been determined [13]. And eighth edition AJCC cancer staging manual firstly defined positive lymph nodes (PLNs) = 1–3 as N1 category and PLNs ≥ 4 as N2 category [13], but there was some controversy about the effect of the number of PLNs on the prognosis of DCC patients [10, 14–16]. The end of this study was to explore the optimal cut-off values of the number of ELNs and PLNs to better stratify patients by utilizing a population-based database.

## Materials and methods

### Database and patients

The data in this study was obtained from the Surveillance, Epidemiology, and End Results (SEER) database, which was one of the most representative large tumor databases in North America.

A number of 817 DCC patients who underwent primary surgical treatment from January 2010 to December 2015 were identified from the SEER*Stat 8.3.6 Software (https://seer.cancer.gov/data/) (S1 Table). The selected database was the Incidence—SEER 18 Regs Custom Data (with additional treatment fields), Nov 2018 Sub (1975–2016 varying). A total of 59 patients whose TNM stage (7th AJCC) were unknown and those who died within 1 month were excluded. Eventually, 758 DCC patients were enrolled in this study.

The data was including the following: (i) general demographic information: gender, age, race; (ii) clinicopathologic information: tumor size, tumor grade, the 7th AJCC stage, lymphadenectomy (number of lymph nodes removed ≥ 1), number of ELNs and PLNs, adjuvant radiotherapy, adjuvant chemotherapy, and follow-up data.

### Statistical analysis

Continuous variables and categorical variables were presented as median (range) and frequency (percentage). Disease-specific survival (DSS) rate was measured from the time of diagnosis to the death of DCC or the last follow-up (November 2018). Survival analysis was comparing by the Kaplan-Meier method (log-rank test). Univariate and multivariate analysis was comparing by Cox proportional hazard regression model, and all variables related to survival (P < 0.2) in univariate analysis were enrolled in multivariate analysis. The X-tile software (https://medicine.yale.edu/lab/rimm/research/software/) was used to determine the optimal cut-off value for the number of ELNs and PLNs. Survival analysis was used for comparing the prognostic stratification ability of the optimal cut-off values of the number of ELNs and PLNs and the recommendation of ELNs (12) and PLNs (0, 3) by the AJCC. A P < 0.05 was considered statistically significant. Statistical analysis was using STATA 16.0 software.

## Results

### Baseline characteristics

This study included 758 DCC patients who underwent primary surgical treatment from January 2010 to December 2015 (Table 1). With a median follow-up of 19 (2–83) months, 420 patients (55.4%) died for DCC, and the 1-, 2- and 3-year DSS rates were 78.8%, 53.2% and 40.0%, respectively.

**Table 1:** Demographics and clinical characteristics in the entire cohort (n = 758).

Demographics	Distal cholangiocarcinoma (n = 758)
Age at diagnosis (years)	68(30–92)
Gender, male/female (%)	475(62.7)/283(37.3)
Race, n (%)	
White	565(74.5)
Black	56(7.4)
Other	137(18.1)
Tumor size (mm)	21(1–118)
Tumor grade, n (%)	
well differentiated	82(10.8)
moderately differentiated	353(46.6)
poorly differentiated	266(35.1)
undifferentiated	6(0.8)
Unkown	51(6.7)
Lymph node metastasis, yes/no (%)	375(49.5)/382(50.5)
Distant metastasis, yes/no (%)	36(4.7)/722(95.3)
AJCC stage (7th), n (%)	
IA	87(11.5)
IB	106(14.0)
IIA	169(22.3)
IIB	333(43.9)
III	27(3.6)
IV	36(4.7)
Lymphadenectomy, yes/no (%)	714(94.2)/44(5.8)
Adjuvant radiotherapy, yes/no or unkown (%)	244(32.2)/514(67.8)
Adjuvant chemotherapy, yes/no or unkown (%)	427(56.3)/331(43.7)

The other comprises American Indian/Alaska Native, Asian/Pacific Islander; AJCC, American Joint Committee on Cancer.

### Prognostic factors

In univariate analysis, tumor size, tumor grade, 7th AJCC T stage, lymph node metastasis, and distant metastasis were associated with prognosis (all P < 0.005, Table 2). Multivariate analysis showed tumor size (HR: 1.322, 95% CI: 1.066–1.639, P = 0.011), tumor grade (HR: 0.744, 95% CI: 0.602–0.921, P = 0.007), 7th AJCC T stage (HR: 0.763, 95% CI: 0.597–0.976, P = 0.031), lymph node metastasis (HR: 1.258, 95% CI: 1.008–1.570, P = 0.042), distant metastasis (HR: 1.643, 95% CI: 1.042–2.591, P = 0.032) as independent predictors (Table 2).

**Table 2:** Univariate and multivariate analysis of prognostic factors for 758 DCC patients.

Factors	N	Univariate		Multivariate	
		3-year DSS rate(%)	P value	HR(95%CI)	P value
Age(years)					
>65	456	39.5	0.416	NA	NA
≤65	302	40.8			
Gender					
male	475	39.4	0.810	NA	NA
female	283	41.0			
Race					
white	565	37.7	0.091	1.265(0.987–1.621)	0.064
black and other	193	46.7			
Tumor size(cm)					
>2	345	33.8	<0.001	1.322(1.066–1.639)	0.011
≤2	334	47.1			
Tumor grade					
well/moderately differentiated	435	44.0	0.001	0.744(0.602–0.921)	0.007
poorly differentiated /undifferentiated	272	32.7			
7th AJCC T stage					
T1, T2	266	51.4	<0.001	0.763(0.597–0.976)	0.031
T3, T4	492	34.1			
Lymph node metastasis					
yes	376	26.2	<0.001	1.258(1.008–1.570)	0.042
no	381	43.9			
Distant metastasis					
yes	36	26.2	0.029	1.643(1.042–2.591)	0.032
no	722	40.5			
Lymphadenectomy					
yes	714	40.5	0.433	NA	NA
no	44	31.5			
Adjuvant radiotherapy					
yes	244	36.1	0.973	NA	NA
no	514	42.0			
Adjuvant chemotherapy					
yes	427	40.7	0.253	NA	NA
no	331	39.0			

DSS, disease-specific survival; HR, hazard ratio; CI, confidence interval; NA, not available; The other comprises American Indian/Alaska Native, Asian/Pacific Islander; AJCC, American Joint Committee on Cancer.

### Exploration of the best cut-off value of number of examined lymph nodes

**Fig 1:**
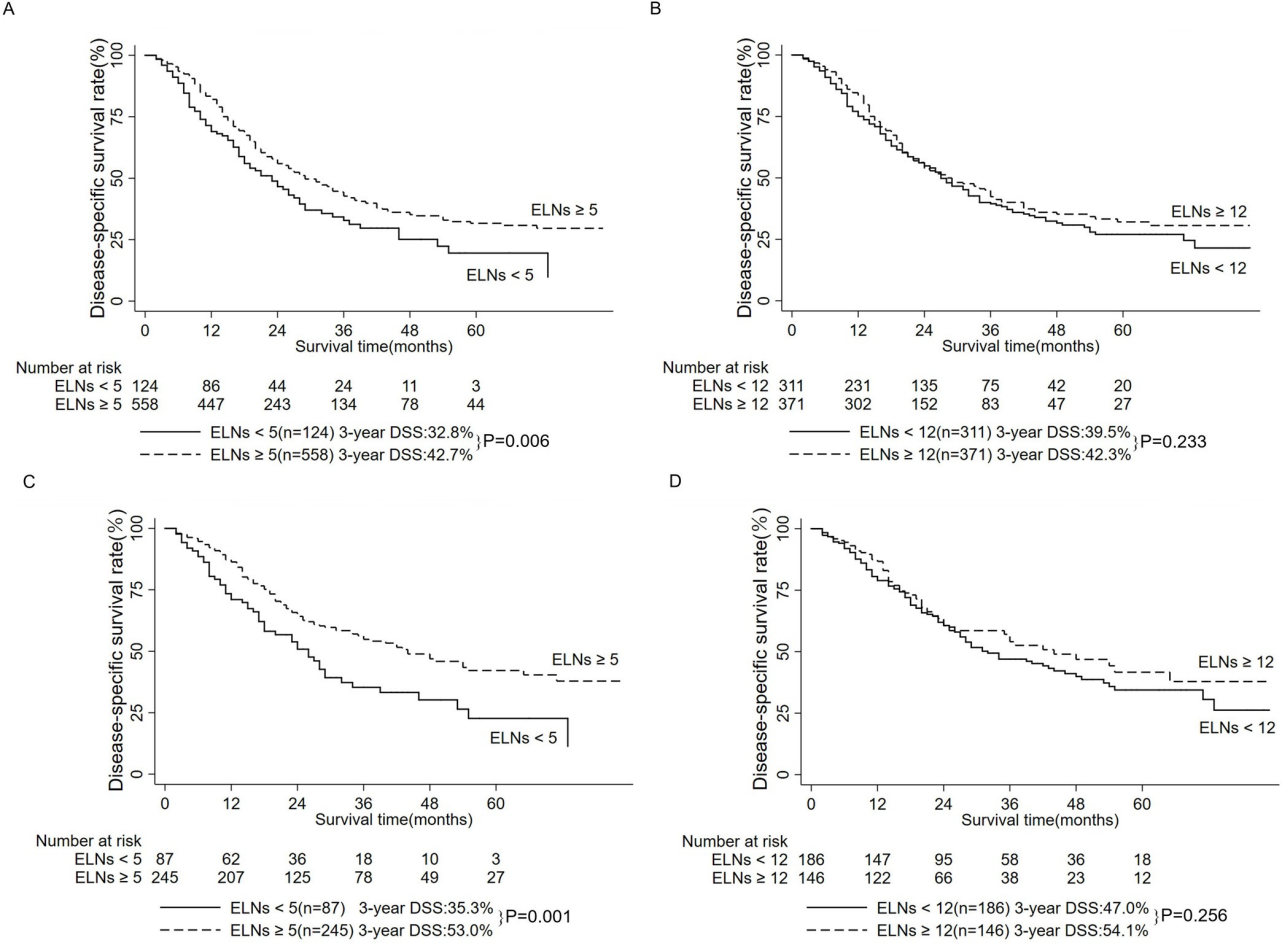
Survival analysis comparing the different numbers of ELNs. A: Survival analysis comparing the optimal cut-off value of the number of ELNs (5) in M0 cohort (n = 682). B: Survival analysis comparing the recommendation of the number of ELNs by the AJCC (12) in M0 cohort (n = 682). C: Survival analysis comparing the optimal cut-off value of the number of ELNs (5) in N0M0 cohort (n = 332). D: Survival analysis comparing the recommendation of the number of ELNs by the AJCC (12) in N0M0 cohort (n = 332).

Among 682 patients with ELNs ≥ 1 in M0 cohort, the best cut-off value of ELNs by X-tile software was 5. Survival analysis found that patients with ELNs < 5 had a lower 3-year DSS rate than those with ELNs ≥ 5 in M0 cohort (32.8% vs. 42.7%, P = 0.006, Fig 1A); survival curves between patients with ELNs < 12 and ELNs ≥ 12 were overlapped in M0 cohort (P = 0.233, Fig 1B).

Besides, among 332 patients with ELNs ≥ 1 in N0M0 cohort, the best cut-off value of ELNs by X-tile software was also 5. Survival analysis found that patients with ELNs < 5 had a lower 3-year DSS rate than those with ELNs ≥ 5 in N0M0 cohort (35.3% vs. 53.0%, P = 0.001, Fig 1C); Survival curves between patients with ELNs < 12 and ELNs ≥ 12 were overlapped in N0M0 cohort (P = 0.256, Fig 1D).

### Exploration of the best cut-off value of the number of positive lymph nodes

The optimal cut-off values of the number of PLNs obtained by X-tile software were all 0 and 2 in three cohorts (682 M0 patients with ELNs ≥ 1, 558 patients with ELNs ≥ 5 and 371 patients with ELNs ≥ 12). The three cohorts of patients were grouped according to the optimal cut-off value of PLNs (0, 2) and the recommendation of PLNs by the AJCC (0, 3), respectively. The DSS rates of the grouped patients were compared by the survival analysis.

**Fig 2:**
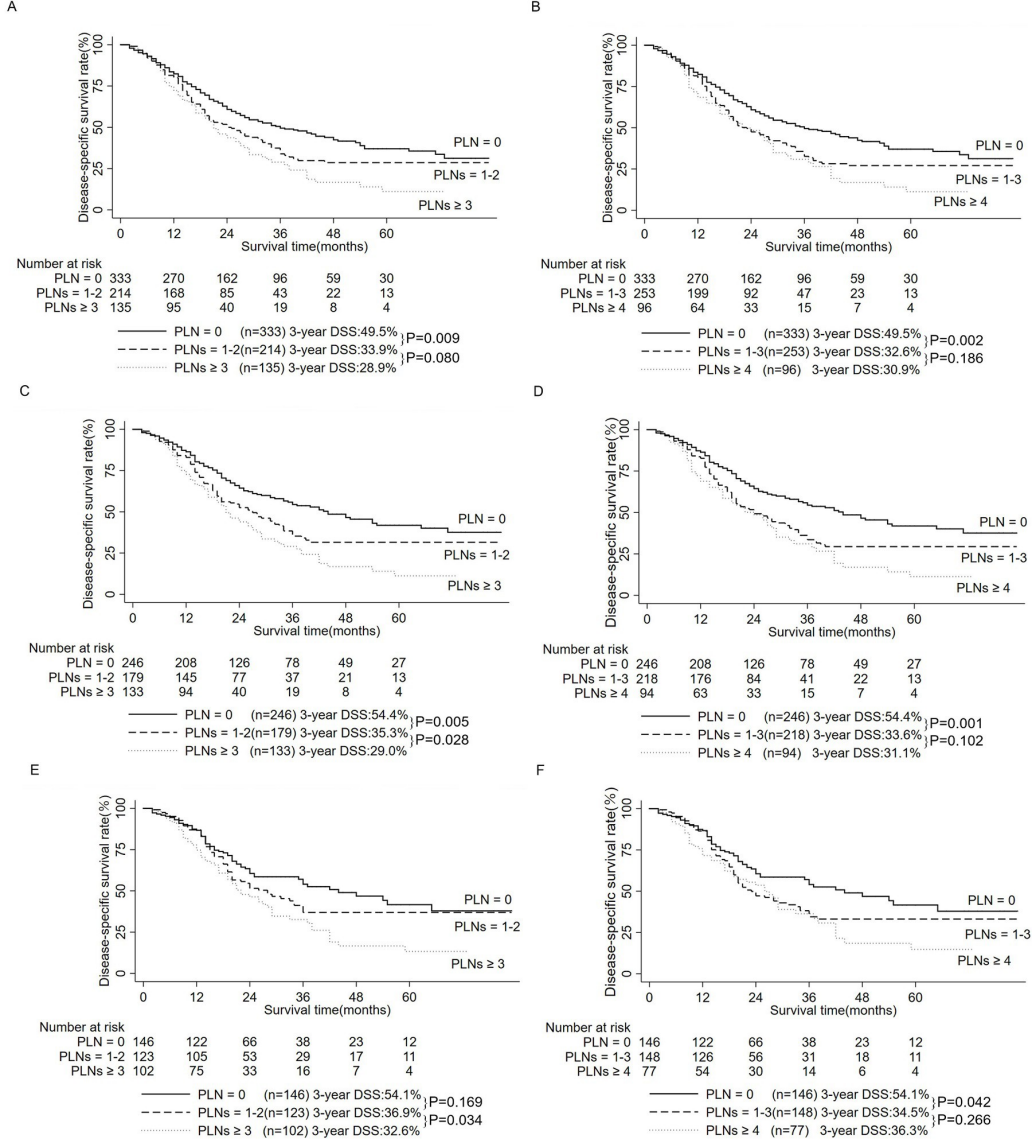
Survival analysis comparing the different numbers of PLNs. A: Survival analysis comparing the optimal cut-off value of the number of PLNs (0, 2) in M0 patients with ELNs ≥ 1 (n = 682). B: Survival analysis comparing the recommendation of the number of PLNs by the AJCC (0, 3) in M0 patients with ELNs ≥ 1 (n = 682). C: Survival analysis comparing the optimal cut-off value of the number of PLNs (0, 2) in M0 patients with ELNs ≥ 5 (n = 558). D: Survival analysis comparing the recommendation of the number of PLNs by the AJCC (0, 3) in M0 patients with ELNs ≥ 5 (n = 558). E: Survival analysis comparing the optimal cut-off value of the number of PLNs (0, 2) in M0 patients with ELNs ≥ 12 (n = 371). F: Survival analysis comparing the recommendation of the number of PLNs by the AJCC (0, 3) in M0 patients with ELNs ≥ 12 (n = 371).

Among 682 patients with ELNs ≥ 1 in M0 cohort, survival analysis found that patients with PLN = 0 had a higher 3-year DSS rate than PLNs = 1–2 (49.5% vs. 33.9%, P = 0.009, Fig 2A), no significant difference in 3-year DSS rates was witnessed between patients with PLNs = 1–2 and PLNs ≥ 3 (33.9% vs. 28.9%, P = 0.080, Fig 2A), and patients with PLN = 0 had a higher 3-year DSS rate than PLNs ≥ 3 (49.5% vs. 28.9%, P < 0.001); The 3-year DSS rate of patients with PLN = 0 was higher than PLNs = 1–3 (49.5% vs. 32.6%, P = 0.002, Fig 2B), no significant difference in 3-year DSS rates was witnessed between patients with PLNs = 1–3 and PLN ≥ 4 (32.6% vs. 30.9%, P = 0.186, Fig 2B), and patients with PLN = 0 had a higher 3-year DSS rate than PLNs ≥ 4 (49.5% vs. 30.9%, P < 0.001).

Among 558 patients with ELNs ≥ 5 in M0 cohort, survival analysis found that patients with PLN = 0 had a higher 3-year DSS rate than PLNs = 1–2 (54.4% vs. 35.3%, P = 0.005, Fig 2C), patients with PLNs = 1–2 had a higher 3-year DSS rate than PLNs ≥ 3 (35.5% vs. 29.0%, P = 0.028, Fig 2C), and patients with PLN = 0 had a higher 3-year DSS rate than PLNs ≥ 3 (54.4% vs. 29.0%, P < 0.001); Patients with PLN = 0 had a higher 3-year DSS rate than PLNs = 1–3 (54.4% vs. 33.6%, P = 0.001, Fig 2D), no significant difference in 3-year DSS rates was witnessed between patients with PLNs = 1–3 and PLNs ≥ 4 (33.6% vs. 31.1%, P = 0.102, Fig 2D), and patients with PLN = 0 had a higher 3-year DSS rate than PLNs ≥ 4 (54.4% vs. 31.1%, P < 0.001).

Among 371 patients with ELNs ≥ 12 in M0 cohort, survival analysis found that no significant difference in 3-year DSS rates was witnessed between patients with PLN = 0 and PLNs = 1–2 (54.1% vs. 36.9%, P = 0.169, Fig 2E), patients with PLNs = 1–2 had a higher 3-year DSS rate than PLNs ≥ 3 (36.9% vs. 32.6%, P = 0.034, Fig 2E), and the 3-year DSS rate of patients with PLN = 0 was higher than those with PLNs ≥ 3 (54.1% vs. 32.6%, P < 0.001); Patients with PLN = 0 had a higher 3-year DSS rate than PLNs = 1–3 (54.1% vs. 34.5%, P = 0.042, Fig 2F), no significant difference in 3-year DSS rates was witnessed between patients with PLNs = 1–3 and PLNs ≥ 4 (34.5% vs. 36.3%, P = 0.266, Fig 2F), and patients with PLN = 0 had a higher 3-year DSS rate than PLNs ≥ 4 (54.1% vs. 36.3%, P = 0.004).

## Discussion

Adequate lymph node dissection could not only improve the prognosis of patients with DCC, but also better stratify the patients. The AJCC recommended that at least 12 lymph nodes be retrieved, whereas the minimum ELNs have not yet been determined [13]. In addition, the 8th AJCC cancer staging manual firstly proposed optimal cut-off value for the number of PLNs of 0 and 3, and it defined N stage as N0, N1, and N2 categories [13]. However, there was some controversy about the effect of number of PLNs on the prognosis for postoperative patients with DCC [10, 14–16]. The aim of this study was to investigate the impact of ELNs and PLNs on the prognosis of DCC patients with resectable disease.

This study recommended examining at least 5 lymph nodes. Although the lymph node dissection was a routine procedure in DCC patients in the European Society for Medical Oncology (ESMO) Clinical Practice Guidelines [17], there was currently some controversy regarding the minimum ELNs [12, 13, 18, 19]. The results of this study found that whether in the N0M0 cohort or the M0 cohort, examining at least 5 lymph nodes could improve the prognosis of patients with DCC (Fig 1A and 1C), but whether the number of ELNs was greater than 12 had nothing to do with the prognosis (Fig 1B and 1D). Furthermore, in patients with ELNs ≥ 12, neither the optimal cut-off value of the number of PLNs (0, 2), nor the recommendation of PLNs by AJCC (0, 3) failed to efficiently stratify patients (Fig 2E and 2F). In contrast, among patients with ELNs ≥ 5, using the optimal cut-off value of the number of PLNs (0, 2) could accurately stratify patients (Fig 2C). Several studies suggested a minimum ELNs of 12 for DCC patients, but they were small in sample size [12, 20]. Moreover, a large sample study from the SEER database found that the prognosis of DCC patients with ELNs = 4–9 was better than other patients [11]. But these studies did not consider the effect of the number of ELNs on the number of PLNs [11, 12, 20]. In summary, this study recommended examining at least 5 lymph nodes to better stratify patients and improve the accuracy of the N staging.

This study suggested that defining PLNs = 1–2 as N1 category may improve the prognostic stratification of patients in the eighth edition of AJCC staging. Nearly half of the patients in this study (49.7%) had lymph node metastasis, which was within the range previously found (44.4–58%) [15, 16]. Lymph node metastasis was significantly associated with poor prognosis, consistent with previous findings [2, 18, 21]. The DCC patients with PLNs were prone to intrahepatic metastases and relapse [22, 23], which will further lead to poor prognosis. Kiriyama M *et al*. [14] found that the number of PLNs was a strong predictor of survival for patients with DCC. Thus, the 8th AJCC staging defined PLNs = 1–3 as N1 category and ≥ 4 as N2 category [13]. Nonetheless, there was some controversy about the effect of the number of PLNs on the prognosis of patients. Firstly, in Kiriyama M *et al*.’s paper [14], patients with PLNs ≥ 4 were only 10% (37/370), and the patients from Japan was selected, which may not be suitable for European and American countries. Secondly, two large sample studies found no difference in prognosis between patients with PLNs ≥ 4 and 1–3 [10, 24], which was consistent with the results of this study. Thirdly, we found that among patients with ELNs ≥ 5, using the optimal cut-off value of PLNs (0, 2) could accurately stratify patients (Fig 2C), but the recommendation of the number of PLNs by the AJCC (0, 3) could not (Fig 2D). Fourthly, previous articles also partly supported the results of this study, and reported that patients with PLNs ≥ 3 were associated with poor prognosis [15, 16]. Hence, this study supported defining PLNs = 1–2 as N1 category.

This study also had several limits. Firstly, this study was limited due to its retrospective nature, and it was necessary to validate our results in large sample prospective studies. Secondly, several clinical features that have a significant impact on prognosis were lacking due to the SEER database, such as surgical margins, tumor recurrence, detailed plans for chemotherapy and radiotherapy, and complications. Thirdly, the number of lymph nodes retrieved may depend on the lymph node dissection technique of each institution, and the hospital type was not clear. Fourthly, there may be some errors in the data from the SEER database. But the SEER database, as a large national database, provided extensive universality for the results of the study.

### Conclusions

This study recommended examining at least 5 lymph nodes and defining PLNs = 1–2 as the N1 category and PLNs ≥ 3 as the N2 category,which may better stratify DCC patients and improve the accuracy of the eighth edition AJCC staging.

## Supporting information

S1 Table Searching data. (DOCX)
