# Supplementary material for: Proposal of the optimal numbers of examined and positive lymph nodes to the 8th edition of American Joint Committee on Cancer (AJCC) staging for 758 patients with distal cholangiocarcinoma
Source: PLoS One. 2020 Jun 16;15(6):e0234464. doi: 10.1371/journal.pone.0234464 (PMC7297328; doi:10.1371/journal.pone.0234464)
Supplement: S1 Table — (DOCX) [file pone.0234464.s001.docx]

A number of 817 DCC patients who underwent primary surgical treatment were identified by that year of diagnosis was from 2010 to 2015, surgery of primary site was encoded as 30-90 (such as simple/partial surgical removal of primary site, total surgical removal of primary site, and radical surgery), primary site was extrahepatic bile duct (C24.0), site recode/WHO 2008 was encoded as extrahepatic bile duct, CS site-specific factor (SSF) 25 was encoded as 40 and 70 (CS-SSF 25 was Schema Discriminator: BileDuctsDistal/BileDuctsPerihilar/CysticDuct, codes 40 and 70 represented distal bile ducts), diagnostic confirmation was positive histology, behavior recode for analysis was malignant, and type of follow-up expected was active follow-up.
